# Simulated Obstructive Sleep Apnea Increases P-Wave Duration and P-Wave Dispersion

**DOI:** 10.1371/journal.pone.0152994

**Published:** 2016-04-12

**Authors:** Thomas Gaisl, Annette M. Wons, Valentina Rossi, Daniel J. Bratton, Christian Schlatzer, Esther I. Schwarz, Giovanni Camen, Malcolm Kohler

**Affiliations:** 1 Department of Pulmonology, University Hospital Zurich, Zurich, Switzerland; 2 Spital Zollikerberg, Zollikerberg, Switzerland; 3 Centre for Integrative Human Physiology, University of Zurich, Zurich, Switzerland; 4 Centre for Interdisciplinary Sleep Research, University of Zurich, Zurich, Switzerland; Charité - Universitätsmedizin Berlin, GERMANY

## Abstract

**Background:**

A high P-wave duration and dispersion (Pd) have been reported to be a prognostic factor for the occurrence of paroxysmal atrial fibrillation (PAF), a condition linked to obstructive sleep apnea (OSA). We tested the hypothesis of whether a short-term increase of P-wave duration and Pd can be induced by respiratory manoeuvres simulating OSA in healthy subjects and in patients with PAF.

**Methods:**

12-lead-electrocardiography (ECG) was recorded continuously in 24 healthy subjects and 33 patients with PAF, while simulating obstructive apnea (Mueller manoeuvre, MM), obstructive hypopnea (inspiration through a threshold load, ITH), central apnea (AP), and during normal breathing (BL) in randomized order. The P-wave duration and Pd was calculated by using dedicated software for ECG-analysis.

**Results:**

P-wave duration and Pd significantly increased during MM and ITH compared to BL in all subjects (+13.1ms and +13.8ms during MM; +11.7ms and +12.9ms during ITH; p<0.001 for all comparisons). In MM, the increase was larger in healthy subjects when compared to patients with PAF (p<0.05).

**Conclusion:**

Intrathoracic pressure swings through simulated obstructive sleep apnea increase P-wave duration and Pd in healthy subjects and in patients with PAF. Our findings imply that intrathoracic pressure swings prolong the intra-atrial and inter-atrial conduction time and therefore may represent an independent trigger factor for the development for PAF.

## Introduction

Obstructive sleep apnea (OSA) is highly prevalent, with approximately 20% of males and 10% of females in Western countries affected by asymptomatic OSA. Pathophysiologically, the disease is characterized by repetitive partial or complete obstruction of the pharynx during sleep. Despite increasing breathing efforts the upper airway collapse results in episodes of obstructive hypopneas or apneas resulting in negative intrathoracic pressure, intermittent hypoxia, autonomic nervous dysfunction, and consequently adverse effects for sleep-architecture and health.[1–3]

A large body of evidence suggests that OSA is associated with cardiac arrhythmias such as paroxysmal atrial fibrillation (PAF) with some studies proposing a causal relationship.[2] Compared with non-OSA patients, severe OSA patients have four times the odds of PAF even after adjustment for potential confounders.[4] The underlying mechanisms of heart rhythm disturbances induced by respiratory events is rather complex and represents a challenge in the context of integrative human physiology. Recent studies suggested that the acute effects of obstructive apneas may distend the atria and may change electrophysiology (especially conduction times), ultimately leading to PAF.[5–7]

Inhomogeneous atrial depolarization secondary to insults such as atrial distension or autonomic nervous dysfunction can be quantified with a surface electrocardiography (ECG)-derived P-wave analysis.[8] A high P-wave duration and P-wave dispersion (Pd) have been reported to be a prognostic factor for the onset of PAF.[8] Moreover, the prolongation of electromechanical delay and an increased Pd are associated with the severity of OSA.[9–12] Because treatment of OSA with continuous positive airway pressure (CPAP) provides a more homogenous conduction (i.e. shorter P-wave durations and less Pd), a causal effect of OSA leading to an increased risk of PAF development is possible.[13,14]

Currently, it is unclear whether hypopneas and apneas have an instant effect on P-wave duration and Pd and whether these pathophysiological events might subsequently alter atrial electrophysiology. Thus we aimed to study this important pathophysiological link and investigate the acute effects of simulated OSA on p-wave indices in healthy subjects and patients with PAF.

## Materials and Methods

The study was approved by the “Cantonal Ethics Committee Zurich”, Switzerland (KEK-ZH-Nr. 2012–0310 / EK 1672) and written informed consent was obtained from all participants.

### Study population

57 patients (24 healthy subjects and 33 patients with PAF) aged between 18 and 75 years were recruited at the study site (University Hospital Zurich, Switzerland). The subjects overlap with two previously published studies and sample size estimation was adopted accordingly.[5,6] In short, these studies investigated the hypothesis whether simulated obstructive apnea could promote arrhythmias e.g. atrial premature or ventricular premature beats. [5,6] Patients with episodes of AF during the examination, therapy with class III anti-arrhythmics (e.g. amiodarone or dronedarone), severe structural heart disease, history of atrial fibrillation ablation therapy or lung transplantation, mental or physical disability precluding informed consent or compliance with the protocol were excluded.

### Breathing manoeuvres

The protocol for breathing manoeuvres simulating hypopneas and apneas (and therefore OSA) which was applied in this study has been described previously.[5,6,15,16] In short, the following breathing manoeuvres were performed in randomized order for 20s each: (i) inspiration through an inspiratory threshold load device (ITH) to generate a threshold pressure of -20mmHg[15] (Threshold IMT, Respironics, NJ, USA) simulating an hypopnea; (ii) a Mueller manoeuvre (MM) to maintain a target intrathoracic pressure of -30 mmHg[15] simulating an obstructive apnea; (iii) a voluntary end-expiratory central apnea (AP); and (iv) a period of normal breathing which served as baseline (BL). A 60s wash-out phase between the manoeuvres was used to ensure that the P-wave duration and Pd were similar at the beginning of each manoeuvre. For the analysis a standardized time-frame of 50s was recorded for each manoeuvre lasting from -10s until +40 s before and after the onset of the manoeuvre, respectively. This 50 second time-frame was also divided into five 10s sectors in which the effect of the breathing manoeuvres on P-wave duration was assessed separately.

### Electrocardiography

Digital 12-lead-ECG was recorded continuously in awake 24 healthy subjects and 33 patients with PAF in supine position (AT 104 PC, Schiller-Reomed AG, Zurich, Switzerland). Room temperature and lighting were set at the same level for all measurements and all subjects remained in supine position five minutes before undergoing assessments. The beginning of the P-wave was defined as the point where the first atrial deflection crossed the isoelectric line and the end of the P-wave was defined as the point where the atrial deflection returned to the isoelectric line. The maximum and minimum P-wave durations in all 12 ECG-leads were retrieved and the most extreme ones were used to calculate the Pd (Pd = maximum P-wave duration − minimum P-wave duration).[8] For comparisons Pd and P-wave duration was averaged from four heartbeats at the first 10 seconds of the manoeuvre where the biggest change in electrophysiology was expected according to previous studies.[6] We explored P-wave duration in a single lead (II or V5) over the 50 second period of each breathing manoeuvre. All ECG recordings were analysed offline by two investigators (TG, AMW) who were blinded to the manoeuvre being performed using dedicated software for ECG-analysis[5] (DatInf Measure 2.1d, DatInf GmbH, Tubingen, Germany).

### Statistical analysis

Continuous data were summarized as mean±SD and categorical data summarized using percentages. The primary outcomes of this study were the mean P-wave duration and Pd during each breathing manoeuvre. Mixed-effects linear regression with patient-level random-intercepts was used to compare the effect of each breathing manoeuvre on each outcome compared to BL. All analyses were adjusted for manoeuvre, heart rate, BMI, age, sex, and PAF status. An interaction between manoeuvre and PAF status was added to each model to compare the effects of each manoeuvre in patients with and without PAF. Mixed-effects linear regression was also used to assess the effect of each manoeuvre on Pd and P-wave duration measured in a single lead over time by including an interaction between manoeuvre and sector.

In order to make sure our measurements were reproducible two blinded investigators each measured a total of 180 P-waves from 20 randomly chosen sectors in which all breathing manoeuvres were performed. Inter-observer agreement was assessed using the intra-class-correlation coefficient and a Bland-Altman plot. The validity of Pd (which represents only the two most extreme values) was checked by assessing its correlation with the standard deviation (which takes values from all 12 leads into account) of all corresponding P-wave measurements. A two-sided p-value less than 0.05 was considered to be statistically significant for all tests. All statistical analyses were performed with STATA version 14 (StataCorp LP, College Station, TX).

## Results

24 healthy subjects and 33 patients with PAF were included in this study. Clinical characteristics of the study population are presented in Table 1. Compared to the healthy population, the PAF patients were on average older (p<0.001), sleepier and tended to be more overweight (Table 1).

**Table 1:** Clinical characteristics by study population.

	Population		
Characteristic	Healthy (n = 24)	PAF (n = 33)	p-value
Age (years)	32.5 (9.9)	61.1 (11.1)	<0.001
Male (%)	18 (75)	28 (85)	0.35
Height (cm)	177.2 (8.9)	176.0 (9.5)	0.63
Weight (kg)	72.5 (12.1)	82.3 (16.5)	0.012
BMI (kg/m^2^)	23.0 (2.7)	26.5 (4.4)	<0.001
Neck Circ. (cm)	36.3 (3.9)	40.1 (4.1)	0.001
Waist/Hip ratio	0.9 (0.1)	1.0 (0.1)	<0.001
ESS	5.6 (2.7)	8.6 (3.6)	0.001
RR interval (ms)	954.3 (125.6)	1033.7 (212.1)	0.083

Data summarized as mean (SD) for continuous variables and N (%) for categorical variables. Continuous variables compared between studies using unpaired t-test. Binary variables compared between studies using χ^2^-test.

BMI = body-mass-index. ESS = Epworth Sleepiness Scale. PAF = paroxysmal atrial fibrillation. RR interval = time from onset of one R wave to the onset of the next R wave in the ECG.

### P-waves

Overall results for Pd and P-wave duration are shown in Table 2. ITH and the MM significantly increased Pd by over 10ms when compared with normal breathing (both p<0.001) in all subjects (n = 57). ITH (-20 mmHg negative intrathoracic pressure) increased Pd by +12.9 ms (95% CI 10.3, 15.6) and the MM (-30 mmHg negative intrathoracic pressure) by +13.8 ms (95% CI 11.2, 16.5) when compared with BL. The P-wave duration was significantly higher during ITH (+11.7 ms; 95% CI 9.3, 14.1) and MM (+13.1 ms; 95% CI 10.8, 15.5) when compared to BL (both p<0.001). The AP manoeuvre was not associated with significant changes compared to BL in P-wave duration (-0.1 ms vs BL; p = 0.90) or Pd (+0.4 ms vs BL; p = 0.79).

**Table 2:** Differences in P-wave mean and Pd in ms during breathing manoeuvres in all patients (n = 57).

	P-wave duration				P-wave dispersion			
Manoeuvre	Mean (SD)	Adjusted difference v BL	95% CI	p-value	Mean (SD)	Adjusted difference v BL	95% CI	p-value
BL	113.9 (10.9)	-	-	-	27.3 (7.4)	-	-	-
ITH	126.1 (11.4)	11.7	9.3, 14.1	<0.001	40.3 (7.9)	12.9	10.3, 15.6	<0.001
MM	126.2 (14.5)	13.1	10.8, 15.5	<0.001	41.0 (8.0)	13.8	11.2, 16.5	<0.001
AP	114.3 (10.2)	-0.1	-2.5, 2.2	0.90	27.7 (6.6)	0.4	-2.3, 3.1	0.79

The ITH-manoeuvre and the MM showed a significant increase in P-wave duration and Pd when compared to BL (normal breathing). No changes occurred in the AP manoeuvre. All analyses were adjusted for PAF status, heart rate, age, BMI, and sex.

BL = baseline (normal breathing). ITH = inspiration through a threshold load (simulating an obstructive hypopnea). MM = Mueller manoeuvre (simulating an obstructive apnea). AP = end-expiratory breath holding (simulating central apnea).

**Fig 1:**
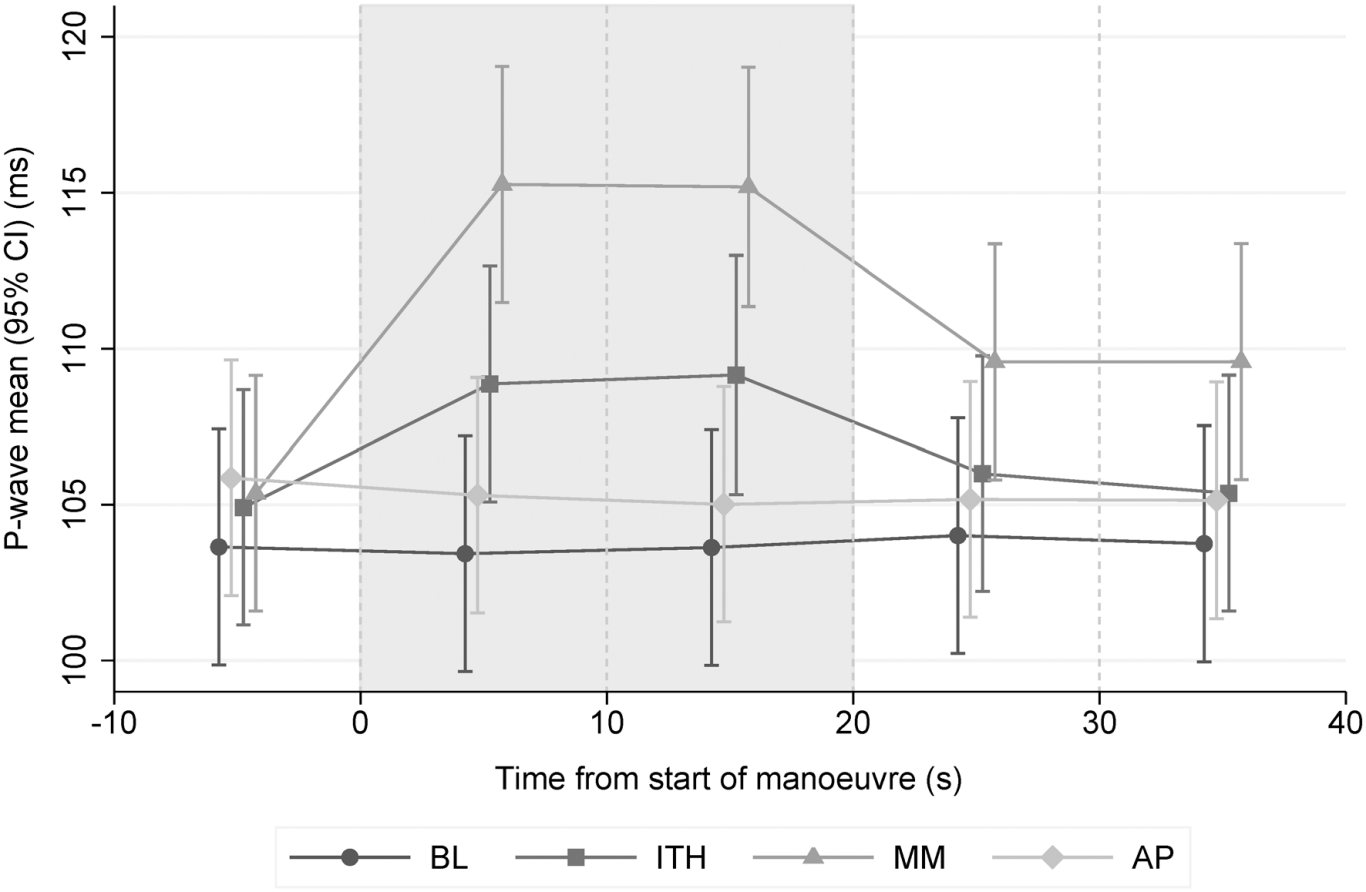
Mean P-wave duration measured in a single lead over all sectors (95% CI). The two sectors which comprise the manoeuvres are shaded grey. We observed significant differences for ITH and MM vs BL during the manoeuvre (both p<0.001). The P-wave duration did not change during the course of the BL (p = 0.99) and AP (p = 0.94) manoeuvre. All analyses were adjusted for PAF status, heart rate, age, BMI, and sex. BL = baseline (normal breathing). ITH = inspiration through a threshold load (simulating an obstructive hypopnea). MM = Mueller manoeuvre (simulating an obstructive apnea). AP = end-expiratory breath holding (simulating central apnea).

Fig 1 displays the mean P-wave duration in a single lead in each manoeuvre and each sector. The P-wave duration did not change during the course of the BL (p = 0.99) and AP (p = 0.94) manoeuvres. By contrast, there were significant increases in P-wave duration during the ITH and MM manoeuvres which dissipated once each manoeuvre ended with much greater increases in the MM. Correlations coefficients between mean P-wave SD and mean Pd were: BL = 0.98, ITH = 0.96, MM = 0.93, AP = 0.94.

### Study populations

**Fig 2:**
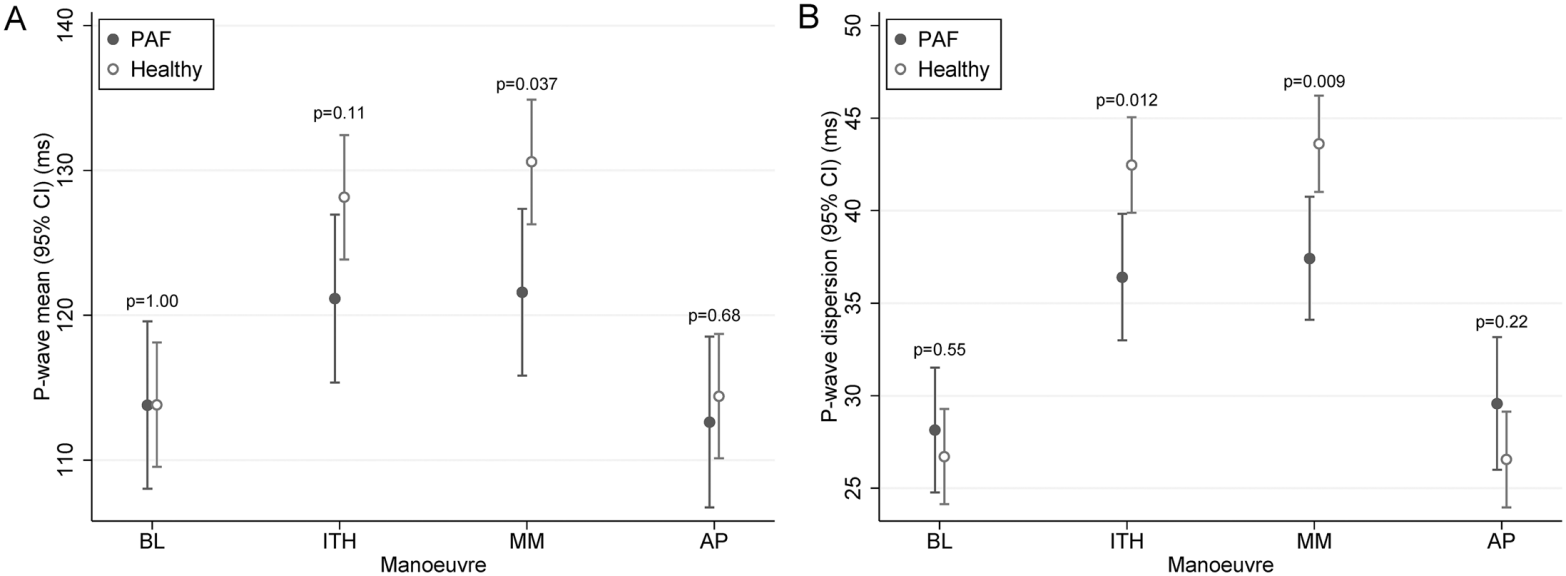
Differences in P-wave mean and Pd during simulated obstructive sleep apnea in healthy subjects (“Non AF”, n = 24) and patients with paroxysmal atrial fibrillation (AF, n = 33). The increase in P-wave duration (**A**) and Pd (**B**) during the MM was significantly higher in patients with PAF when compared to healthy subjects. No significant changes were observed between the two study populations during BL and the AP manoeuvre. All analyses were adjusted for heart rate, age, BMI, and sex. BL = baseline (normal breathing). ITH = inspiration through a threshold load (simulating an obstructive hypopnea). MM = Mueller manoeuvre (simulating an obstructive apnea). AP = end-expiratory breath holding (simulating central apnea).

None of the healthy subjects reported usage of cardio-active medication. Patients with PAF reported use of beta-blocker (82%), other blood pressure lowering agents (57%), and anti-arrhythmic agents (27%). During normal breathing (BL), patients with PAF did not differ from healthy subjects in P-wave duration (p = 1.00) and Pd (p = 0.55) after adjustment for heart rate, age, BMI, and sex. Despite no differences in BL, non-AF individuals were more susceptible to P-wave duration increases (p = 0.037) and Pd-increases (p = 0.009) during the MM than patients with PAF. P-wave durations and Pd for AF and non-AF participants during each manoeuvre are presented in Fig 2.

### Inter-observer agreement

The intra class correlation coefficient was 0.96 (95% CI 0.95–0.98) and the 95% limits of agreement were -4.49 ms / 5.10 ms showing very high inter-observer agreement in P-wave measurements. For further information see S1 Appendix.

## Discussion

This study is, to our knowledge, the first to investigate the acute effects of simulated apnea and hypopnea on P-wave duration and Pd, markers of atrial conduction delay and inhomogeneous atrial depolarization. This study provides data to better understand the pathophysiological mechanisms that may be involved in patients with OSA and the onset of PAF. The presented data demonstrates how simulated obstructive hypopnea (ITH) and apnea (MM) increases P-wave duration and Pd and thus might have a direct effect on arrhythmogenesis. Our findings that P-wave duration and Pd are increased in patients with PAF are in line with several other studies in literature.[8]

### Clinical implications

The findings of this study may have some important implications for patients at risk for PAF. A large body of evidence suggests that OSA is linked to PAF via mechanisms like elevated blood pressure and intermittent hypoxia among others.[2] This study, however, supports evidence that the instant features of OSA directly influence markers of cardiac arrhythmic potential. P-wave duration has been shown to be highly correlated with intra-atrial conduction times.[17] It is known that patients with OSA feature a disturbed P-wave morphology and P-wave duration and Pd have been shown to correlate with the apnea-hypopnea index, a marker of disease severity.[9] Animal models have shown that apneas and hypopneas themselves distend cardiac structures and alter electrophysiology.[18] This has been confirmed in several studies conducted in humans.[5,6] Pd can be used to assess the individual propensity to PAF onset.[8] This study links the pathophysiology of OSA to altered P-wave morphology and shows how the immediate effects of OSA have an impact on P-wave morphology. At this point, the possibility that a significant proportion of the adult population in Western countries may be affected by asymptomatic OSA should be kept in mind. Further research is warranted to assess the long-term relationship between ECG-derived risk markers and the onset of PAF in cohorts with OSA patients. Surface ECG-derived markers represent an elegant and cheap diagnostic tool and it has been advocated to include P-wave indices in automated ECG-reports.

Recent research suggests that the success rate of PAF treatment (i.e. pulmonary vein isolation) is dependent on the diagnosis of OSA. Meta-analyses have shown that patients with OSA have significantly greater AF recurrence rates after pulmonary vein isolation and CPAP-treatment can reverse this excess risk.[19] ECG-derived risk markers might have the potential to accurately predict PAF recurrence after ablation therapy in patients with OSA.[8] However, this needs to be proven in future studies focused on this point.[8]

### Potential mechanisms

Generally the mechanisms leading to the pathogenesis of PAF remain complex and a matter of debate. It is thought that atrial distortion and atrial enlargement are central to the development of PAF.[7] In our previous work, we demonstrated that instant negative intrathoracic pressure changes contribute to cardiac dysrhythmias in healthy humans and in patients with PAF.[5,6] Second, acute and chronic dysfunction in the autonomic nervous system associated with OSA lead to cellular mechanisms of myocardial adaptation possibly contributing to PAF.[20] Autonomic nervous system dysfunction severity (especially sympathetic over-activity) caused by OSA on myocardial depolarization (i.e. QRS duration) is a significant clinical marker in terms of the ventricular arrhythmic risk in OSA patients.[21] In fact, data from our study might support a potential autonomic mechanism given that PAF patients (of whom 82% were on beta-blockers) were less susceptible to simulated OSA manoeuvres in Pd and P-wave duration. In addition, augmented oxidative stress has been suggested to increase the risk of cardiac arrhythmias.[22] In our study we could rule out any detrimental effects of instant hypoxia with the AP-manoeuvre, based on previous experience with the manoeuvre[6]. Thus the authors hypothesize, that intrathoracic pressure changes and autonomic nervous system dysfunction are important mechanisms in patients with OSA contributing to atrial electrical and structural remodeling, ultimately leading to the onset of PAF.

According to catheter measurements, approximately 85% of the airway pressure drop is transmitted into the atria.[23] The physical effect of atrial stretch paired with autonomic nervous dysfunction several hundred times per night might induce chronic atrial changes resulting in remodeling and functional burden in patients with untreated OSA.[7] This mechanism may be modifiable with CPAP-treatment; however this seems not to be the case with oxygen therapy.

### Strengths and limitations

The use of digital measurements, the high inter-observer agreement between blinded investigators, and the interchangeability between Pd and the SD of the measurements generate high confidence in the validity of the data. Compared with other studies in this field, the present one scores above average in these parametes.[8]

Given the design of this pathophysiological study it is important to emphasize that artificially induced changes in breathing patterns were evaluated which could limit the clinical relevance of the data. However, the physiology of the breathing manoeuvres in our study were validated using esophageal and aortic catheters[15] the authors feel confident that simulated apneas reflect real life conditions and the results can be generalized to patients with OSA.[24] Moreover, our team has gathered considerable experience in mimicking real-life conditions for hypopneas and apneas.[5,6,15,16] However, we did not directly measure sympathetic activity which might influence cardiac arrhythmic risk[21] and OSA status (i.e. sleep studies) was not assessed in this study. Whether previous exposure to obstructive events might affect the response of apneas on atrial conduction remains to be elucidated. Changes in autonomic nervous system induced by simulated OSA in awake patients might also differ significantly from changes during different sleep stages (e.g. rapid eye movement) which potentially limits the generalizability of the data. Although, we excluded hypoxia (AP) as a potential mechanism in our study[6], it is very difficult to distinguish the contribution of the role of atrial stretch versus autonomic nervous activity and further research in this area is warranted.

### Conclusion

In conclusion this study provides evidence that breathing manoeuvres simulating obstructive sleep apnea promote an immediate increase of P-wave duration and Pd and provide a potential explanation for the increased risk of PAF in patients with OSA. Our findings imply that intrathoracic pressure swings prolong the intra-atrial conduction time and therefore could be an independent trigger factor for the development for PAF.

## Supporting Information

S1 Appendix Inter-observer agreement. (PDF)
