# Supplementary material for: Simulated Obstructive Sleep Apnea Increases P-Wave Duration and P-Wave Dispersion
Source: PLoS One. 2016 Apr 12;11(4):e0152994. doi: 10.1371/journal.pone.0152994 (PMC4829247; doi:10.1371/journal.pone.0152994)
Supplement: S1 Appendix — (PDF) [file pone.0152994.s001.pdf]

Supporting information

Simulated Obstructive Sleep Apnea Increases P-wave Duration and P-wave Dispersion

Thomas Gaisl[1], Annette M. Wons[1], Valentina Rossi[1], Daniel J. Bratton[1], Christian Schlatzer[1], Esther I. Schwarz[1], Giovanni Camen[1], Malcolm Kohler[1,2,3]

[1]Department of Pulmonology, University Hospital Zurich, Zurich, Switzerland

[2]Centre for Integrative Human Physiology, University of Zurich, Zurich, Switzerland

[3]Centre for Interdisciplinary Sleep Research, University of Zurich, Zurich, Switzerland

Corresponding author information:

Professor Malcolm Kohler, Chair of Respiratory Medicine, Clinical Director Division of Pulmonology, University Hospital Zurich, Raemistrasse 100, 8091 Zurich, Switzerland.

E-mail: Malcolm.Kohler@usz.ch

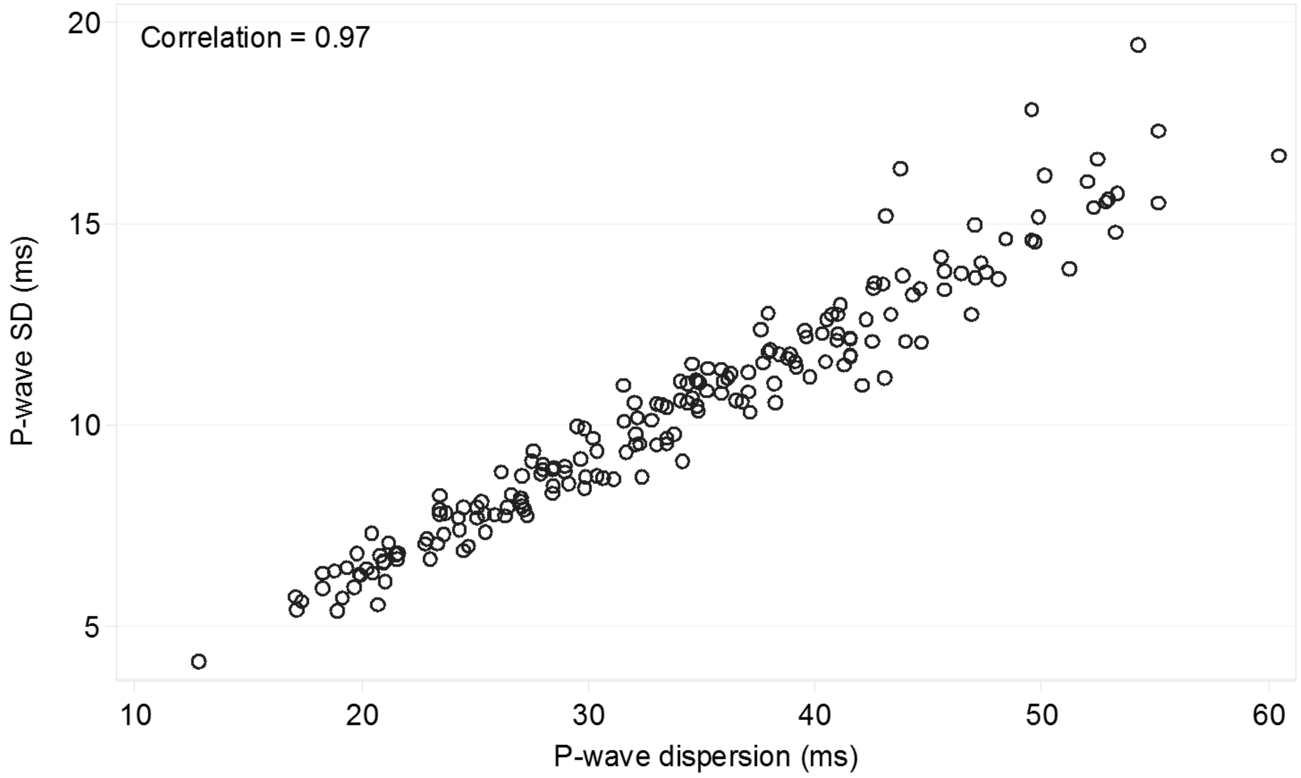

Correlation between p-wave dispersion over all manoeuvres and p-wave SD. The maximum and minimum p-wave durations in all 12 ECG-leads were retrieved and the most extreme ones were used to calculate the Pd (Pd = maximum p-wave duration − minimum p-wave duration). The overall correlation coefficient was 0.96.

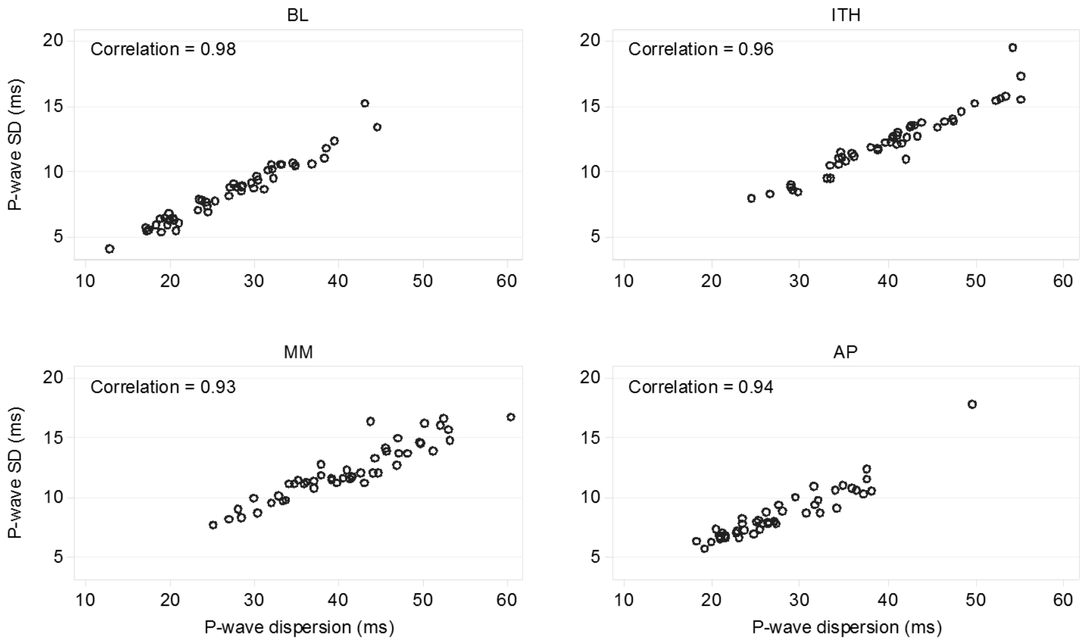

Correlation between p-wave dispersion within each manoeuvre (p max – p min in all 12 ECG leads) and p-wave SD. Correlations coefficients between mean p-wave SD and mean Pd were: BL = 0.98, ITH = 0.96, MM = 0.93, AP = 0.94. BL = baseline (normal breathing). ITH = inspiration through a threshold load (simulating an obstructive hypopnea). MM = Mueller manoeuvre (simulating an obstructive apnea). AP = end-expiratory breath holding (simulating central apnea).

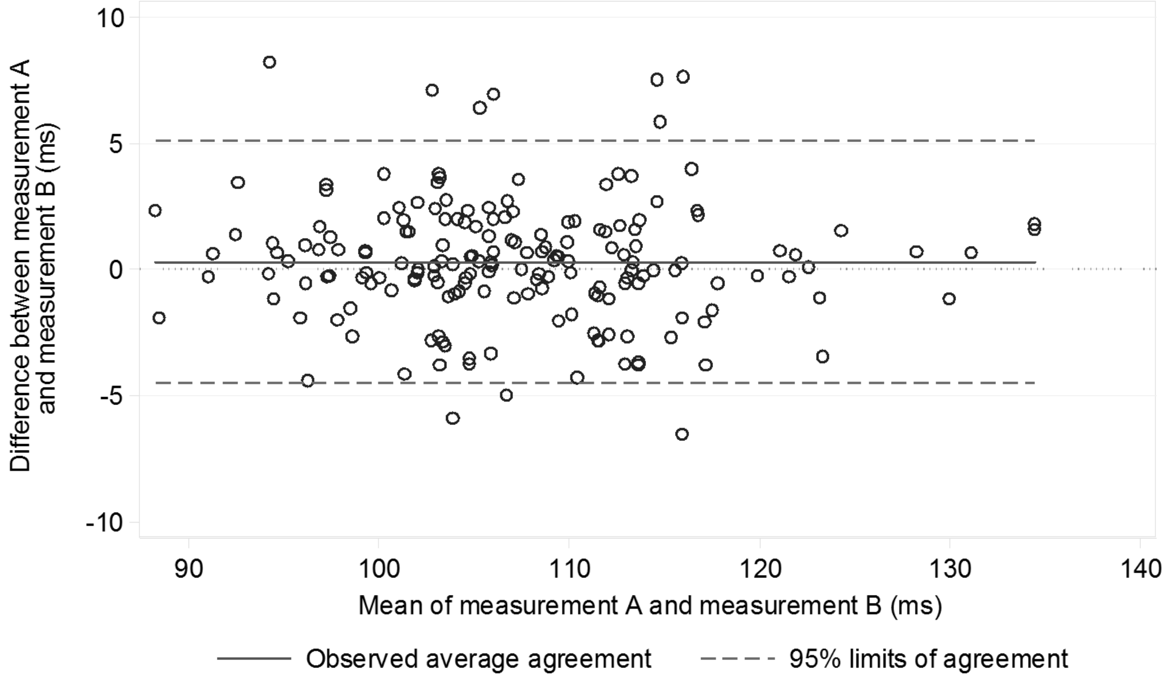

Bland-Altman plot for 180 p-waves measured by two investigators (A and B). y=0 is line of perfect average agreement. The 95% limits of agreement were -4.49 ms / 5.10 ms.

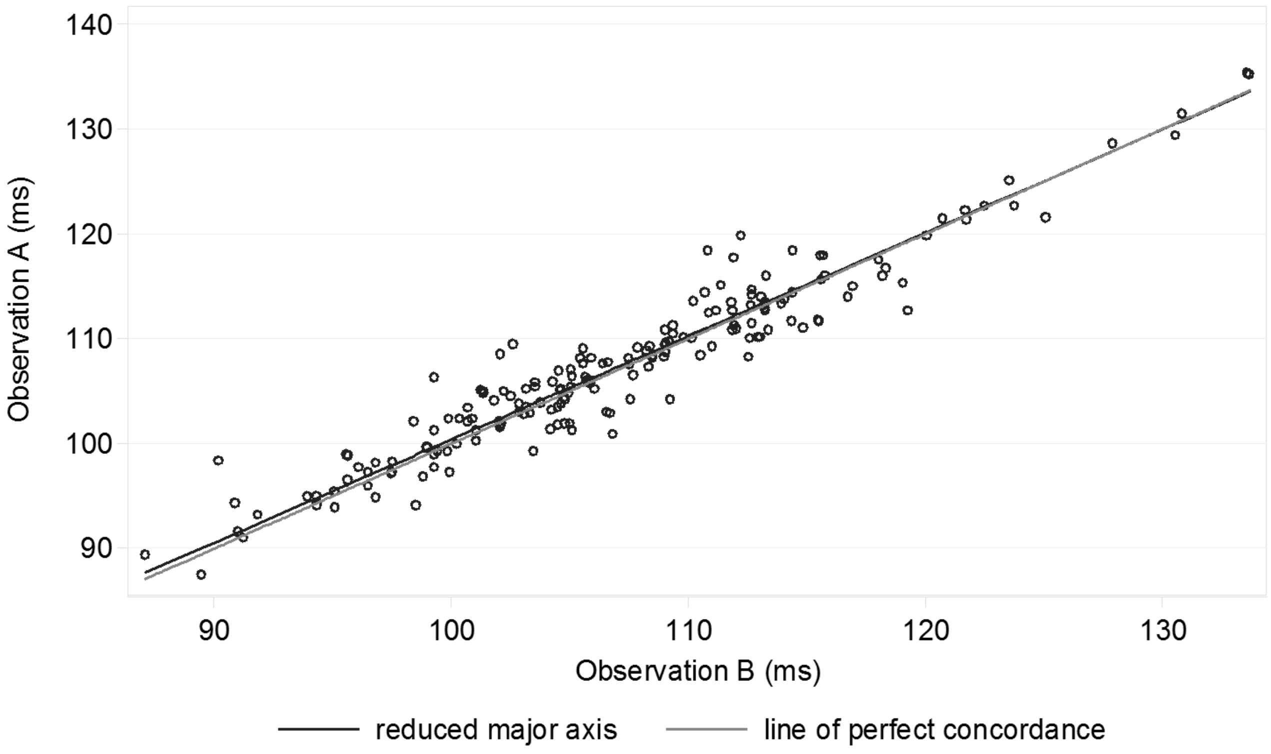

Graphical display of data between investigators. Graphical display of data and reduced major axis between observation A (original measurement) and observation B (re-measured by blinded investigator).
